# Arthritis as an initial presentation of malignancy: two case reports

**DOI:** 10.1186/s13256-020-02642-z

**Published:** 2021-02-23

**Authors:** Benjamin Sachdev Manjit Singh, Sharifah Aishah Wan, Yaw Kiet Cheong, Seow Lin Chuah, Cheng Lay Teh, Ahmad Tirmizi Jobli

**Affiliations:** 1 Rheumatology Unit, Department of Medicine, Sarawak General Hospital, Kuching, Malaysia; 2 Department of Radiology, Faculty of Medicine and Health Sciences, University Malaysia Sarawak, Kota Samarahan, Malaysia

**Keywords:** Paraneoplastic, Arthritis, Malignancy

## Abstract

**Background:**

Arthritis is rarely reported as a paraneoplastic manifestation of occult malignancy. We report herein two cases of paraneoplastic arthritis due to occult malignancy.

**Case 1:**

The patient was a 65-year-old woman of asian descent who was a former smoker with a history of spine surgery performed for L4/L5 degenerative disc disease. She presented with a 1-month history of oligoarthritis affecting both ankle joints and early morning stiffness of about 3 hours. Laboratory tests were positive for antinuclear antibody at a titer of 1:320 (speckled) but negative for rheumatoid factor. She was treated for seronegative spondyloarthritis and started on prednisolone without much improvement. A routine chest radiograph incidentally revealed a right lung mass which was found to be adenocarcinoma of the lung. She was treated with gefitinib and her arthritis resolved.

**Case 2:**

The patient was a 64-year-old woman of asian descent, nonsmoker, who presented with a chief complaint of asymmetrical polyarthritis involving her right wrist, second and third metacarpophalangeal joints, and first to fifth proximal interphalangeal joints. She was treated for seronegative rheumatoid arthritis (RA) and started on sulfasalazine, with poor clinical response. Six months later, she developed abdominal pain which was diagnosed as ovarian carcinoma by laparotomy. Her arthritis resolved following treatment of her malignancy with chemotherapy.

**Conclusion:**

In summary, paraneoplastic arthritis usually presents in an atypical manner and responds poorly to disease-modifying antirheumatic drugs. Accordingly, we recommend screening for occult malignancy in patients presenting with atypical arthritis.

## Background

Paraneoplastic syndromes are a collection of signs and symptoms caused by organ or tissue damage occurring at locations distant from primary tumors [1]. These symptoms may develop before, concurrent with, or after the diagnosis of a malignancy [2]. Interestingly, malignancies have been associated with a wide variety of paraneoplastic rheumatic manifestations which may develop in joints, fascia, muscles, vessels or bones, often mimicking the presentation of primary rheumatic diseases such as spondyloarthropathy (SpA), rheumatoid arthritis (RA), systemic lupus erythematosus (SLE) or vasculitis [2]. We report two patients whose cases were initially diagnosed and treated as rheumatic diseases but were later found to be paraneoplastic arthritis related to malignancy.

## Case presentation

### Case 1

**Fig. 1:**
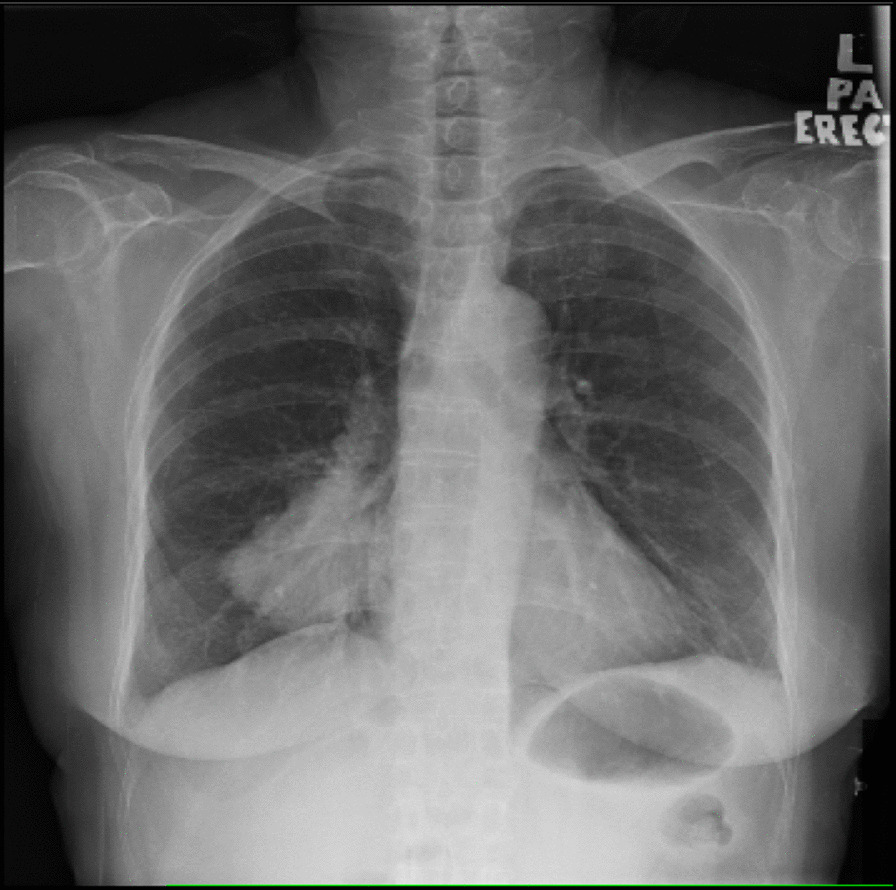
Chest X-ray (posteroanterior erect view) with mass over the right lower lobe

**Fig. 2:**
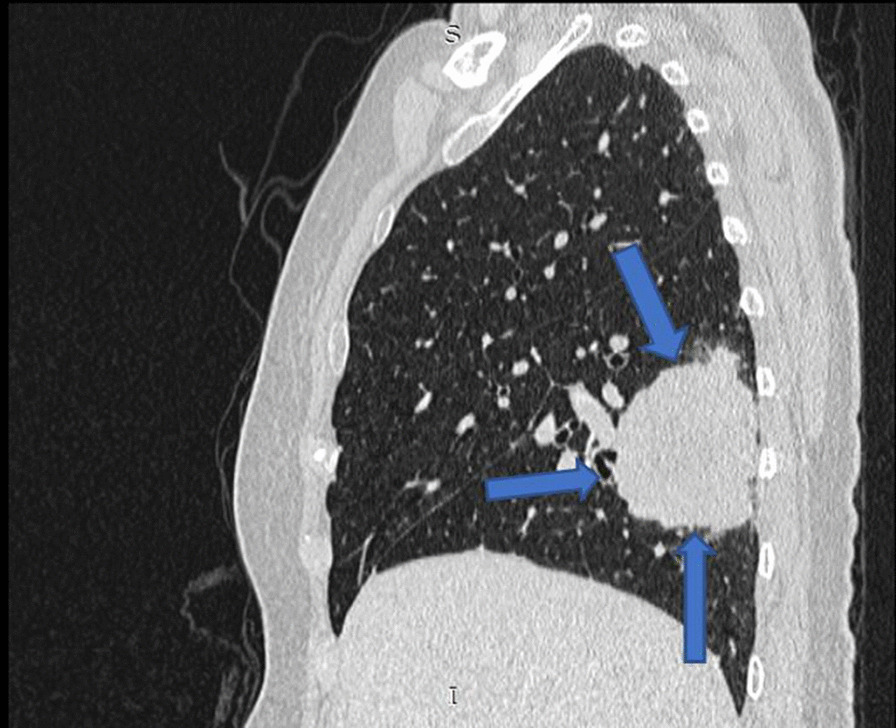
Sagittal computed tomography scan of the thorax demonstrates mass at the right lower lobe with associated surrounding satellite nodules (arrows)

The patient was a 65-year-old woman of asian descent who was a former smoker for 20 years and had a history of spine surgery for L4/L5 degenerative disc disease. She was a retiree and a nondrinker. She presented with a 1-month history of oligoarthritis affecting both ankle joints associated with early morning stiffness of about 3 hours. On physical examination, the patient had swelling and tenderness of both ankle joints, with no other significant findings. She had no significant family history of malignancy or inflammatory arthritis. Laboratory tests were negative for rheumatoid factor (RF) but positive for antinuclear antibody (ANA) at a titer of 1:320 (speckled pattern). Erythrocyte sedimentation rate (ESR) was 61 mm/hour. Accordingly, she was treated for SpA, for which she was started on prednisolone 10 mg once daily and sulfasalazine (SSZ) 500 mg twice a day, without much improvement. Her SSZ was however withheld during a later clinic visit, as she developed macular rash after her dosage was increased to 1 g once daily. A routine chest radiograph (Fig. 1) performed on her first visit incidentally revealed a right lung mass. Subsequent contrast-enhanced computed tomography (CT) of the thorax (Fig. 2) showed a lung mass at the posterobasal segment of the right lower lobe measuring 4.7 × 7.0 × 7.0 cm (anteroposterior × width × craniocaudal). There were also satellite nodules adjacent to the mass. The mass was biopsied via bronchoscopy, and histopathological examination (HPE) results showed an adenocarcinoma favoring a primary lung malignancy, which showed a deletion in exon 19 of the epidermal growth factor receptor (EGFR) gene. Therefore, she was treated with tablet gefitinib 250 mg daily by the oncology team. The swelling of both ankles resolved with the initiation of gefitinib for her malignancy, with no recurrence on subsequent follow-up visits to the rheumatology clinic over the next 6 months. She was able to ambulate without any difficulty or pain. Her final diagnosis was revised to paraneoplastic arthritis secondary to adenocarcinoma of the lung. She was discharged to continue follow-up under the oncology team for her malignancy.

### Case 2

**Fig. 3:**
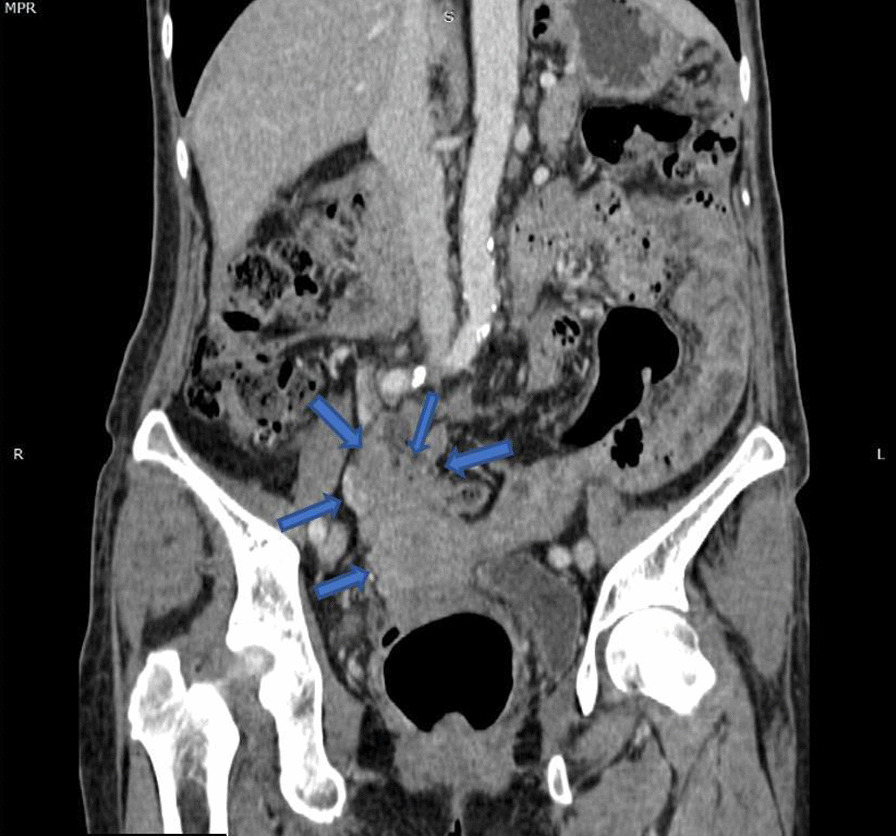
Coronal computed tomography scan image demonstrating uterine mass infiltrating the adjacent bowels and omentum (blue arrows)

**Fig. 4:**
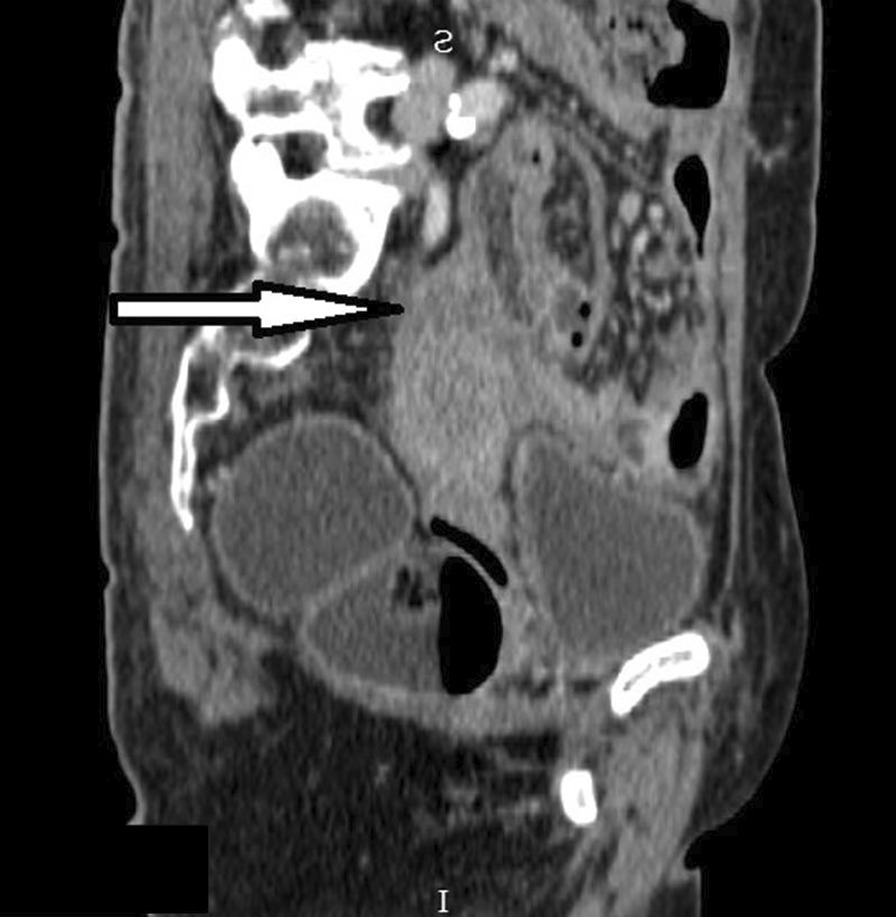
Sagittal computed tomography scan image demonstrating uterine fundus mass which has poor demarcation with the uterus inferiorly (arrow)

The patient was a 64-year-old woman of asian descent who was a nonsmoker, nondrinker and known to have hypertension, diabetes mellitus, dyslipidemia and ischemic heart disease. She was a retiree and was previously on the following medications: metformin 500 mg once daily, amlodipine 10 mg daily, atenolol 100 mg daily, isosorbide dinitrate 10 mg three times a day, aspirin 75 mg once a day, simvastatin 10 mg once daily and perindopril 2 mg daily. She presented with a 4-month history of asymmetrical polyarthritis involving her right wrist, second and third metacarpophalangeal joints (MCPJ), and first to fifth proximal interphalangeal joints (PIPJ) associated with loss of appetite and loss of weight. She however denied other constitutional symptoms. She had no family history of malignancy or connective tissue disease. Examination revealed swollen and tender joints involving her right wrist, second and third MCPJ and first to fifth PIPJ. There were no other significant physical findings on examination. Her routine laboratory workup was unremarkable aside from a raised ESR and positive antinuclear antibody at a titer of 1:160 (centromere pattern). Rheumatoid factor (RF) was negative, and a routine chest radiograph was unremarkable. X-rays of the hands showed arthritis changes in both hands, possibly RA. She was treated as seronegative RA and started on SSZ 500 mg twice daily and prednisolone 5 mg once daily, with poor clinical response. Six months after her initial arthritis, she presented to a private center with complaints of abdominal pain, resulting in a mini-laparotomy with omental biopsy. The omental HPE was reported as metastatic deposits of grade II serous cystadenocarcinoma of the ovary in the omentum. She was diagnosed with stage IIIc ovarian carcinoma and started neoadjuvant chemotherapy with intravenous administration of carboplatin AUC (area under the curve) 5 at day 1, with paclitaxel 80 mg/m^2^ on days 1, 8 and 15 for six cycles every 21 days. Her SSZ was withheld. After three cycles of chemotherapy, she developed new respiratory symptoms. A repeat CT of the thorax/abdomen revealed a left apico-posterior lung mass with multiple bilateral lung nodules, with an ill-defined hypodense mass at the region of the uterine fundus (Figs. 3, 4). This was followed by a rigid bronchoscopy revealing a pedunculated tumor from the left upper lobe almost completely occluding the distal left main bronchus, which was biopsied. The biopsy showed a malignant tumor with sarcomatoid features. She proceeded to complete the six cycles of carboplatin/paclitaxel. A CT reassessment post-carboplatin/paclitaxel showed disease progression, as evidenced by a larger left lung mass on CT. She was then given second-line chemotherapy consisting of gemcitabine 1400 mg intravenously on day 1 and day 8, planned for three cycles and reassessment of response. Hence, her final diagnosis by the oncology team was dual malignancy (stage IIIc ovarian serous cystadenocarcinoma and malignant tumor with sarcomatoid features of the left lung). During all her follow-up visits with the oncology team she was noted to have no recurrence of her polyarthritis but was poorly responsive to her chemotherapy, with progressive respiratory symptoms. About 1 year after the initial diagnosis of polyarthritis, the patient succumbed to her illness after an admission for pneumonia.

## Discussion

In both patients described above, the symptoms of inflammatory arthritis preceded the detection of malignancy. Our first patient was a 65-year-old woman who presented with oligoarthritis of the ankle joints which was treated as SpA. The second patient presented with asymmetrical polyarthritis, which was treated as seronegative RA. Both our patients had poor clinical response to disease-modifying antirheumatic drugs (DMARDs) and were later found to have malignancy that was adenocarcinoma of the lung in the first patient and ovarian carcinoma in the second patient. In both patients, their arthritis resolved only after therapy for their malignancy was initiated.

Arthritis is a common symptom encountered by the rheumatologist, with a broad range of causes including RA, SpA, vasculitis, connective tissue diseases, crystal arthropathies and infections. It is however an uncommon presentation of paraneoplastic syndrome. Paraneoplastic syndromes, which include paraneoplastic arthritis, may precede or occur during the course of malignant disease [2–4]. They should not arise from direct tumor invasion or compression, and usually improve with treatment of the underlying malignancy [3, 4]. Paraneoplastic arthritis itself is an inflammatory polyarthritis which may be seronegative [5]. It has a male predominance, with an average age of onset of 54 years [5]. The arthritis is typically asymmetrical, commonly affecting the lower extremity joints, and may occur about 8–12 months prior to malignancy development [1, 3–5]. It is usually poorly responsive to the standard therapy of DMARDs, corticosteroids or nonsteroidal anti-inflammatory drugs (NSAIDs) [1, 3–5]. Malignancies associated with paraneoplastic arthritis include lung, breast, ovarian, laryngeal and gastrointestinal tumors [2–4]. The pathogenesis is not known, but mediators such as cytotoxic lymphocytes, antibodies, peptides, hormones and cytokines have been implicated [4, 6]

According to Zupancic *et al.*, paraneoplastic arthritis can be difficult to diagnose in the absence of a known malignancy [4]. This difficulty is illustrated by the two cases discussed above, where the symptoms of arthritis preceded the detection of malignancy. Similar to other reports, both our patients presented with symptoms suggestive of an inflammatory arthritis which were subsequently diagnosed and treated as rheumatic diseases [4]. In the first patient, the malignancy was found following and abnormal routine chest radiograph. Similarly, for the second patient, the malignancy only became apparent after the patient presented with abdominal pain requiring surgical intervention, resulting in the diagnosis of a stage IIIc ovarian carcinoma.

Finally, both our patients exhibited poor response to DMARDs and showed improvement after treatment of their underlying malignancy, leading to the suspicion of paraneoplastic arthritis. This poor response to DMARDs and improvement with antineoplastic therapy was described by both Briones *et al.* and Zupancic *et al.* in two different case reports [3, 4]. Briones described a patient who presented with polyarthritis and responded poorly to glucocorticoid therapy and was subsequently diagnosed with lingual carcinoma. The patient was treated with antineoplastic therapy, leading to progressive resolution of the arthritis [3]. Similarly, Zupancic described a patient with a history of migratory inflammatory asymmetric polyarthritis which responded poorly to prednisolone. The patient was subsequently diagnosed with small cell lung carcinoma, and arthritis symptoms resolved with treatment of the malignancy [4]. Hence, it is important that occult malignancy be suspected as a cause of arthritis in a patient who responds poorly to DMARD therapy.

## Conclusion

In conclusion, paraneoplastic arthritis is a rare initial presentation of malignancy. The presence of arthritis in the absence of a known malignancy can be easily confused with a primary rheumatic condition. This ultimately leads to a delay in the diagnosis of an underlying primary malignancy. Hence, it is important to consider paraneoplastic arthritis in patients who present atypically, develop constitutional symptoms or respond poorly to standard DMARD therapy.

## Data Availability

Data sharing is not applicable to this article as no datasets were generated or analyzed during the current study.

## Abbreviations

SpA: Spondyloarthropathy
RA: Rheumatoid arthritis
SLE: Systemic lupus erythematosus
DMARDs: Disease-modifying antirheumatic drugs
NSAIDs: Nonsteroidal anti-inflammatory drugs
ANA: Antinuclear antibody
RF: Rheumatoid factor
MCPJ: Metacarpophalangeal joints
PIPJ: Proximal interphalangeal joints
ESR: Erythrocyte sedimentation rate
SSZ: Sulfasalazine
HPE: Histopathological examination
CT: Computed tomography
